# Pharmacological and Molecular Insight on the Cardioprotective Role of Apigenin

**DOI:** 10.3390/nu15020385

**Published:** 2023-01-12

**Authors:** Shilu Deepa Thomas, Niraj Kumar Jha, Saurabh Kumar Jha, Bassem Sadek, Shreesh Ojha

**Affiliations:** 1 Department of Pharmacology and Therapeutics, College of Medicine and Health Sciences, United Arab Emirates University, Al Ain P.O. Box 15551, United Arab Emirates; 2 Department of Biotechnology, School of Engineering & Technology (SET), Sharda University, Greater Noida 201310, Uttar Pradesh, India; 3 Department of Biotechnology, School of Applied & Life Sciences (SALS), Uttaranchal University, Dehradun 248007, Uttarakhand, India; 4 School of Bioengineering and Biosciences, Lovely Professional University, Phagwara 144411, Punjab, India; 5 Department of Biotechnology Engineering and Food Technology, Chandigarh University, Mohali 140413, Punjab, India; 6 Zayed Bin Sultan Center for Health Sciences, United Arab Emirates University, Al Ain P.O. Box 15551, United Arab Emirates

**Keywords:** antioxidants, anti-inflammatory, apigenin, cardioprotection, flavonoid, heart, myocardial protection, polyphenolic, polyphenols

## Abstract

Apigenin is a naturally occurring dietary flavonoid found abundantly in fruits and vegetables. It possesses a wide range of biological properties that exert antioxidant, anti-inflammatory, anticancer, and antibacterial effects. These effects have been reported to be beneficial in the treatment of atherosclerosis, stroke, hypertension, ischemia/reperfusion-induced myocardial injury, and diabetic cardiomyopathy, and provide protection against drug-induced cardiotoxicity. These potential therapeutic effects advocate the exploration of the cardioprotective actions of apigenin. This review focuses on apigenin, and the possible pharmacological mechanisms involved in the protection against cardiovascular diseases. We further discuss its therapeutic uses and highlight its potential applications in the treatment of various cardiovascular disorders. Apigenin displays encouraging results, which may have implications in the development of novel strategies for the treatment of cardiovascular diseases. With the commercial availability of apigenin as a dietary supplement, the outcomes of preclinical studies may provide the investigational basis for future translational strategies evaluating the potential of apigenin in the treatment of cardiovascular disorders. Further preclinical and clinical investigations are required to characterize the safety and efficacy of apigenin and establish it as a nutraceutical as well as a therapeutic agent to be used alone or as an adjuvant with current drugs.

## 1. Introduction

Flavonoids are naturally occurring polyphenols with significant biological actions systemically demonstrated in several in vitro and in vivo experimental models. Apigenin, chemically known as 4′,5,7-trihydroxyflavone, is one of the widely distributed flavonoid compounds in the plant kingdom, including fruits, spices, and vegetables. It is present in fruits, such as oranges, vegetables (parsley, celery), herbs (chamomile, thyme), and beverages (tea, beer, and wine) [1]. Apigenin has received widespread attention for its commercial application in the food and flavor industry as well as its beneficial effects in promoting health and wellness, owing to its versatile biological effects that include antitumor, antioxidant, antiapoptotic, and anti-inflammatory properties. These biological effects on different organs (heart, brain, liver, and lung) are well-translated in the therapeutic and preventive potential in numerous diseased conditions, including hypertension, hyperglycemia, hyperlipidemia, osteoporosis, arthritis, and immune regulation [2]. Apigenin exhibits anti-inflammatory responses through the modulation of p38/MAPK, PI3K/Akt as well as NF-κB pathways [3]. Activation of the Nrf2-signaling pathway is also implicated in its cardioprotective effects. Experimental studies have revealed the therapeutic effects of apigenin in various pathological conditions, such as cancer, asthma, diabetes, pancreatitis, insomnia, anxiety, depression, and Alzheimer’s disease [4,5].

Considering the health benefits and dietary availability, efforts have been made in the past few years to promote its nutraceutical usage. Regarding the biopharmaceutic characteristics, it has been classified as a Class II molecule in the Biopharmaceutics Classification System (BCS) owing to the high membrane permeability and poor solubility [6]. BCS plays a role in signifying oral drug absorption, solubility, and intestinal permeability, and is thus considered useful in drug discovery, development and regulatory concerns. The molecules of BCS Class II display differences in their formulation, gastrointestinal solubility, and dissolution rate. The molecules belong to class II and consist of carboxylic acids with a pKa value between 4 and 5 that attributes them with insolubility at a typical, fasted, gastric pH, but makes them soluble at intestinal pH.

In the present review, we comprehensively reviewed the beneficial effects of apigenin, focusing on cardiovascular diseases, including atherosclerosis, stroke, myocardial infarction, diabetic cardiomyopathy, hypertension, cardiac hypertrophy, and aneurysm.

## 2. Sources of Apigenin

Flavonoids are dietary polyphenolic compounds that are of vital importance to human health and have promising prospects as key nutraceuticals. Apigenin is one of the most common flavonoids found in plants and belongs to the flavone subclass [5]. Apigenin is found in chamomile tea, obtained from the dried flowers of *Matricaria chamomilla* (Asteraceae), an annual herb native to Western Asia and Europe. Drinks prepared from chamomile contain 0.8% to 1.2% apigenin. Chamomile tea is one of the most popular herbal teas around the world and widely recognized for its soothing and calming effect. As a traditional medicine, it is used to treat wounds, ulcers, eczema, skin irritations, bruises, burns, neuralgia, sciatica, rheumatic pain, and hemorrhoids [7]. The presence of apigenin in passion flower (*Passiflora incarnata,* Passifloraceae) makes it beneficial for asthma, neuralgia, anxiety, and insomnia [8].

Apigenin has also been found to be an active bioactive molecule in other species, such as *Scutellaria barbata* D. Don (Lamiaceae), *Portulaca oleracea* L. (Portulacaceae), *Marrubium globosum* ssp. *Libanoticum* (Lamiaceae), *Combretum erythrophyllum* (Combretaceae), *Gentiana veitchiorum* (Gentianaceae), and propolis, most of them being used in traditional herbal or alternative medicines [1]. Apigenin is also found in parsley, celery, and oregano, the richest sources being the respective dried forms [8]. In some plants, apigenin is present in the form of aglycone or as several apigenin glycosides. The common apigenin glycosides are apigenin-7-O-glucoside, apigenin-6-C-glucoside (isovitexin), apigenin-8-C-glucoside (vitexin), apigenin-7-O-neohesperidoside (rhoifolin), and apigenin-6-C-glucoside-8-C-arabinoside [4]. Table 1 lists the various dietary sources of apigenin.

## 3. Pharmacological Actions of Apigenin

Several studies explicate prospects for the therapeutic and preventive usage of apigenin against various ailments, including cardiovascular diseases. It has been demonstrated to exert immunomodulatory, antioxidant, and anti-inflammatory effects [11]. Additionally, apigenin also exerts beneficial effects in models of neurological disorders, attributed to its antioxidant and anti-inflammatory actions. It has been found to exert neuroprotective effects against copper-induced Aβ-toxicity in neuronal cells, which is attributed to preserved mitochondrial function, reduction in neuronal apoptosis and regulation of redox imbalance through the inhibition of p38 mitogen activated protein kinase (p38 MAPK) and stress activated protein kinase (SAPK)/c-JNK pathways [12]. The beneficial effects of apigenin have also been demonstrated in animal models of depression and Parkinson’s disease [4].

Various preclinical investigations demonstrated the protective effects of apigenin on type 2 diabetes mellitus (T2DM), evidenced by the mitigation of oxidative stress, reduced insulin resistance, and favorable correction of irregularities in glycolipid metabolism [13]. Jung et al. investigated the therapeutic potential of apigenin in obesity and related metabolic disturbances in a high-fat diet-induced obesity model [14]. Apigenin was also found to lower the plasma levels of free fatty acids, total cholesterol, apolipoprotein B and hepatic injury markers and ameliorated the progression of hepatic steatosis and hepatomegaly. The reduction in endothelial dysfunction along with improvement in insulin resistance further strengthens its therapeutic implications in metabolic disorders [6].

In animal models with acute lung injury and asthma, apigenin exhibits an inhibitory effect on the levels of inflammatory cytokines, including TNF-α, IL-6, and IL-1β as well as reduced eosinophilia in lung and airway tissues [15,16]. It has been found to exert the inhibition of EV71 virus replication through the suppression of viral IRES activity and the modulation of the cellular JNK pathways [17]. Further, it also appears to interfere with the translational activity of the foot-and-mouth disease virus elicited by the internal ribosome entry sites [18]. Its chemopreventive and chemotherapeutic activity has also been observed in cancers of the breast, colon, liver, prostate, pancreas, ovary, and skin. Several studies demonstrate that co-administration of apigenin increases the efficacy of chemotherapeutic agents against cancer cells as well as attenuating organ toxicity induced by chemotherapeutic agents [8].

## 4. Therapeutic Potential of Apigenin in Cardiovascular Disorders

### 4.1. Atherosclerosis

Atherosclerosis is one of the major underlying causes of cardiovascular and cerebrovascular diseases. The pathogenesis of atherosclerosis is complex and multifactorial, involving lipid accumulation, oxidative stress, chronic inflammation, and endothelial dysfunction. Oxidative stress in endothelial cells stimulates multiple biological processes leading to the upregulation of proinflammatory markers, cellular adhesion molecules, and chemokine production [19]. Apigenin is reported to exert protective effects against the development of atherosclerosis mediated by its antioxidative and anti-inflammatory activities.

Apigenin treatment in animal models of hyperlipidemia has been shown to decrease the levels of total cholesterol, triglycerides, and LDL-C in mice as well as mitigating the accumulation of total cholesterol and triglycerides in the liver, which indicates its potential beneficial effects on atherosclerosis and fatty liver disease. This is accompanied by a significant reduction in excessive body weight. Apigenin has been observed to exert a beneficial role in the metabolism of cholesterol and the protection of vascular endothelial cells [20].

In addition, human umbilical vein endothelial cells (EA. hy926 cells) injured by H_2_O_2_ were also shown to benefit from the protective effect of apigenin against oxidization. Apigenin induced SOD activity and NO secretion, which further confirmed its antioxidative capacity and protective effect on vascular endothelial cells. Apigenin has also been found to regulate cholesterol metabolism by upregulating the decreased expression of HMGCR mRNA and CYP7A1 mRNA. The hypolipidemic effect, along with its anti-inflammatory and antioxidant potential, can be therapeutically beneficial to impede the development of atherosclerosis. Furthermore, apigenin decreases the expression of proinflammatory cytokines and increases the apoptosis of foam cells during atherogenesis [21].

The antihyperlipidemic effects of apigenin were further evaluated by Wong et al. using a mouse model of high-fat diet-fed-induced hypercholesterolemia supplemented with apigenin (50 ppm and 250 ppm) [22]. Dietary supplementation with apigenin hinders the increase in plasma lipid and cholesterol induced by high-fat intake. The expression of SREBP-2 protein was reduced with apigenin administration in the diet, while the activation of AMPK was observed [22]. The activation of AMPK by phosphorylation inhibits SREBP activity and cholesterol biosynthesis. The transcriptional factor SREBP-2 regulates the expression of HMGCR, which catalyzes the rate-limiting reaction in the biosynthesis of cholesterol. The suppression of HMGCR and nuclear SREBP-2 activity blocks cholesterol biosynthesis, leading to low plasma cholesterol levels in apigenin-treated mice.

In another study by Wong et al., apigenin (60 and 300 ppm) was co-administered with a high-cholesterol diet in golden hamsters. Consistent with the findings of the previous study, hamsters fed with apigenin showcased a decrease in serum total cholesterol and triglyceride levels [23]. Apigenin co-administered with the diet resulted in a protective effect against aortic plaque formation and promoted the fecal elimination of cholesterol. Apigenin also reduced the mRNA expression of Npc1l1, which interferes with the uptake of cholesterol in the small intestine, whereas an increase in the expression of hepatic LDLR was observed. This results in the enhanced clearance of circulating LDL-C in the plasma.

The administration of apigenin has also been demonstrated to improve hyperlipidemia and reduce plasma TC, TG, LDL-C, and LOX-1, whereas levels of HDL-C were elevated in hyperlipidemic rats. The intragastric administration of apigenin (40 and 80 mg/kg) also suppressed the oxidation of LDL and upregulated the levels of Bcl-2, thereby preventing atherosclerosis [24]. Furthermore, apigenin reduced the expression of LOX-1 and the inflammasome protein, NLRP3, and inhibited the adhesion of U937 cells to human endothelial cells in response to trimethylamine-N-oxide, reducing the expression of adhesion molecules and the uptake of acetylated LDL [25].

The dysregulation of macrophage apoptosis leads to inflammatory atherogenic processes. Zeng et al. studied the antiatherogenic effects of apigenin in apolipoprotein E-deficient (apo𝐸^−/−^) mice. Apigenin induced the apoptosis of OxLDL- loaded murine peritoneal macrophages (MPMs) with the upregulation of proapoptotic Bax, and cleaved caspase-3, whereas the expression of antiapoptotic Mcl-1 and Bcl-2 was suppressed [26]. The upregulation of apoptosis in macrophages contributed to the antiatherosclerotic effects of apigenin. Apigenin also downregulated antiapoptotic PAI-2 in OxLDL-loaded MPMs. Its proapoptotic actions were significantly decreased with augmented PAI-2 expression.

Clayton et al. demonstrated that oral apigenin administration (50 mg/kg) reverses vascular endothelial dysfunction and aortic stiffening with aging in C57BL/6 male mice and mitigates vascular inflammation, thereby reducing the development of age-related cardiovascular diseases [27]. The improvement in endothelial function has been attributed to the restoration of NO bioavailability as well as suppression of arterial ROS production and oxidative stress by apigenin.

Kumar et al. isolated and characterized apigenin from the plant *Justicia gendarussa* (Acanthaceae) and reported its anti-inflammatory activity mediating inhibition of the TLR-NF-κB signaling pathway. Apigenin was showed to exert anti-inflammatory actions in the in vitro studies carried out on hPBMCs (human peripheral blood mononuclear cells) induced with ox-LDL [28]. Pretreatment with apigenin (25 μM) downregulated the levels of TLR4 and several adaptor proteins, such as MyD88, TRIF, and TRAF6. NF-κB levels were also suppressed with apigenin treatment. Activated TLRs recruit adapter proteins MyD88, TRIF, TRAF, etc., within the cytosol, thereby activating signaling pathways and downstream proteins [29].

TLRs play a major role in the immune system and their activation leads to the production of proinflammatory cytokines. TLRs activate the transcription factor, NF-κB through its interaction with adaptor proteins. Activation of NF-κB has been implicated in the pathogenesis of asthma, rheumatoid arthritis, inflammatory bowel disease, cancer, and atherosclerosis. Furthermore, a significant reduction in the production of proinflammatory cytokines (TNF-α, IL-1β) and an increase in the levels of anti-inflammatory cytokines (IL-10) were also observed with apigenin pretreatment. Hence, it regulates both proinflammatory and anti-inflammatory mediators involved in an immune response.

Further, Ren et al. demonstrated that treatment with apigenin considerably augments ABCA1 expression through miR-33 repression and subsequent rise in ABCA1-mediated cholesterol efflux. ABCA1 prevents excessive lipid accumulation by the removal of cholesterol from lipid-loaded macrophages, which prevents the formation of foam cells and sustains cellular lipid homeostasis [30]. Apigenin was also found to reduce the production of IL-1β, IL-6, and TNF-α in LPS-treated macrophages. The atheroprotective effect of apigenin has been attributed to the favorable suppression of the TLR-4/NF-κB pathway.

Apigenin prevents the translocation of NF-κB into the nucleus through decreasing the phosphorylation of IκB-α, which leads to its anti-inflammatory effects. Upon treatment with apigenin (25 µM), a significant inhibition was reported on the adhesion of THP-1 monocytes (human monocytic cell line, THP-1 cells) to oxidized LDL-activated HUVECs. This effect can be attributed to the downregulation of adhesion molecules, VCAM 1, and E-selectin [31]. This further signifies the potential antiatherogenic actions of apigenin on the oxidized LDL-mediated process associated with atherosclerosis.

### 4.2. Cerebrovascular Diseases

Cerebrovascular diseases are one of the leading causes of death and disability worldwide. Inflammation, apoptosis, and oxidative stress are involved in the pathogenesis of ischemic stroke [32]. Ischemic stroke results in a deficient supply of glucose and oxygen to cerebral tissues resulting in neuronal death. Ischemia-reperfusion injury (IRI) or reoxygenation injury occurs when the blood supply returns to the tissue after a period of hypoxia. The neuroprotective effects of apigenin in vivo have been studied in detail using experimental models of stroke, such as middle cerebral artery occlusion (MCAO) [33,34,35] and the bilateral common carotid artery occlusion (BCCAO) models of cerebral ischemia [33].

Apigenin treatment was found to reduce neurological deficits and infarct volume and improve neuronal survival associated with ischemic stress. Cai et al. evaluated a glycoside subtype of apigenin, apigenin-7-*O*-β-D-(-6″-*p*-coumaroyl)-glucopyranoside, for its neuroprotective effects using three different models of experimental stroke [33]. BCCAO in mice and MCAO in rats and in vitro experiments in the PC12 cell line were the models used for the investigations. Treatment with apigenin (50 and 100 mg/kg, i.p.) considerably improved neurological function. Apigenin attenuated brain damage and improved neuronal survival upon ischemia-reperfusion injury and reduced infarct volume. Apigenin treatment also reduced the levels of MDA, ROS and increased the levels of antioxidant enzymes CAT and SOD, which exert protection against cerebral I/R injury. Furthermore, treatment with apigenin (0.4 μg/mL and 0.8 μg/mL) also improved cell viability, decreased LDH release, and reduced the number of apoptotic cells in PC 12 cell lines.

The upregulation of the caveolin-1/VEGF pathway has also been implicated in the neuroprotective effects of apigenin against cerebral ischemia-reperfusion injury [36,37] by inhibiting apoptosis and autophagy and promoting cell proliferation and migration. Apigenin has been shown to mitigate brain damage and improve neurological deficits in MCAO rats. Caveolin-1 regulates blood–brain barrier permeability, promotes angiogenesis, and plays a key role in cellular repair in stroke, while VEGF protects neurons from ischemic injury and contributes to neurogenesis after cerebral ischemic stroke. Apigenin (40 mg/kg, i.p.) was found to improve post-stroke cognitive impairment through the regulation of histone acetylation and by the upregulation of brain-derived neurotrophic factors [38].

The neuroprotective effect of apigenin has been attributed to its capability to activate the Nrf2 signaling pathway in a Keap-1 independent manner [35]. Apigenin (50 mg/kg, i.p.) treatment stimulated the nuclear translocation of Nrf2, leading to a significant increase in the expression of its target antioxidant genes [35]. Nrf2 is phosphorylated by GSK-3β at a site for the recognition of β-TrCP. Nrf2 ubiquitination and its degradation are regulated by β-TrCP. Hence, GSK-3β negatively regulates Nrf2 and affects its distribution in the cytoplasm and nucleus. Apart from regulating physiological processes, GSK-3β also plays an important role in the development of cellular responses to pathophysiological insults. The phosphorylation of GSK-3β at Ser-9 leads to its inactivation, which exerts protection against cellular damage, whereas dephosphorylation diminishes cellular tolerance to such insults. Treatment with apigenin promoted the phosphorylation of GSK-3β, followed by its inactivation and Nrf2 activation.

Ling et al. further confirmed the beneficial role of apigenin in cerebral ischemia-reperfusion (I/R) injury in PC12 cells exposed to CoCl_2_ as well as in the MCAO model in rats. In both models, apigenin exhibited significant efficacy against cerebral I/R injury [39]. Pretreatment with apigenin improved cellular viability against oxidative stress and injury and lowered ROS production and cellular apoptosis. Apigenin (25 mg/kg) improved functional deficit and reduced the infarct area in rats. In a study by Zhang et al., an apigenin derivative (6’’-O-succcinylapigenin (40 and 60 mg/kg, i.p.) was found to exert neuroprotection following ischemia by regulating the ERK/Nrf2/HO-1 pathway [34]. These results suggest that the apigenin derivative activates the ERK pathway, thereby stimulating the nuclear translocation of Nrf2, leading to anti-inflammatory effects.

Oxidative stress has been convincingly determined to play a critical role in the pathogenesis of subarachnoid hemorrhage. Apigenin exerts a neuroprotective effect through its antioxidant and antiapoptotic activity. Han et al. assessed the protective role played by apigenin in early brain injury following subarachnoid hemorrhage [40]. The administration of apigenin (10 and 20 mg/kg, i.p.) significantly alleviated neurological deficits, brain edema, and blood–brain barrier permeability in rats following subarachnoid hemorrhage. Apigenin treatment decreased the levels of ROS, malondialdehyde (MDA), and myeloperoxidase (MPO), while increasing the levels of glutathione (GSH) and superoxide dismutase (SOD) in the brain cortex.

**Figure 1:**
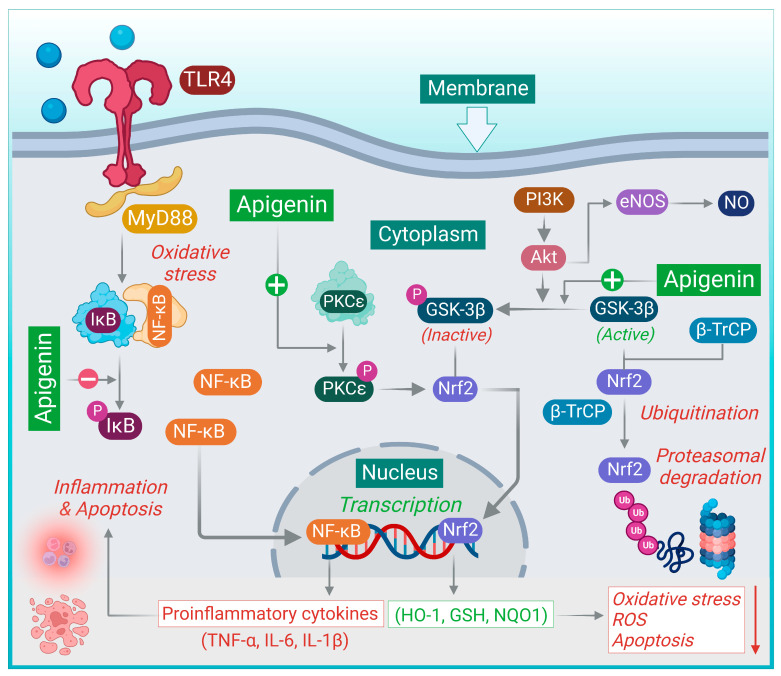
The molecular mechanism of cardioprotective actions of apigenin.

Studies by Zhang et al. demonstrated that apigenin (20 mg/kg, i.p.) attenuates early brain injury and preserves the integrity of the blood–brain barrier by inhibiting the TLR4-mediated inflammatory pathway in subarachnoid hemorrhage [41]. Blood–brain barrier disruption, brain edema, neurological deficiency, and cell apoptosis were reduced considerably after apigenin treatment. Administration of apigenin inhibited TLR4 and NF-κB pathways and consequently reduced TNF-α, IL-6, and IL-1β. Figure 1 summarizes the various molecular mechanisms attributed to the therapeutic effects of apigenin.

Under oxidative stress, TLRs activate the transcription factor, NF-κB through the interaction with the adaptor protein MyD88. Apigenin inhibits phospho-IκB-α and suppresses the activation and nuclear translocation of NF-κB, inhibiting the release of proinflammatory cytokines. Nrf2 is phosphorylated by GSK-3β at a site for the recognition of β-TrCP leading to its proteasomal degradation. Phosphorylation of GSK-3β at Ser-9 leads to its inactivation, attributing protection against cellular damage. Apigenin promotes the phosphorylation of GSK-3β, leading to its inactivation and prevents the degradation of Nrf2. The activation of PKCε by apigenin enhances the nuclear translocation of Nrf2 and subsequent antioxidant pathways. The activation of the PI3K/Akt signaling pathway promotes endothelial cell survival and NO production through the activation of eNOS.

### 4.3. Myocardial Ischemia

Inflammation plays a detrimental role in the progression of myocardial ischemia-reperfusion (I/R) injury. The restoration of perfusion to infarct tissues allows the recovery of the myocardium; however, reperfusion itself may exacerbate ischemic damage and cause myocardial I/R injury [42]. Multiple evidence has shown that apigenin is effective in preventing myocardial I/R associated injury and improving cardiac function. Treatment with apigenin reduces infarct size as well as the activity of LDH and CK [43,44,45]. Apigenin attenuates apoptosis in cardiomyocytes and upregulates antiapoptotic Bcl-2 protein and downregulates Bax, a propoptotic protein. The inhibition of phosphorylation of p38 MAPK and upregulation of Bcl-2 expression contribute to the cardioprotective activity of apigenin.

Apigenin exerts its effects through the inhibition of the NF-κB pathway [46]. Quan and colleagues evaluated apigenin-7-*O*-β-D-(6″-*p*-coumaroyl)-glucopyranoside in an experimental model of myocardial ischemia/reperfusion injury. The administration of apigenin (50 and 100 mg/kg, i.p.) significantly reduced the myocardial infarct size. The myocardial injury enzymes (CK-MB and LDH) as well as proinflammatory cytokines were suppressed with apigenin treatment, resulting in its protective effects. Apigenin treatment also suppressed the levels of the key adhesion molecules involved in inflammation, ICAM-1. Under normal conditions, NF-κB is inactive and present in the cytoplasm as a dimer complexed with the inhibitory protein known as IκB-α. In stressful events, IκB is phosphorylated, leading to the release of NF-κB followed by its nuclear translocation and expression of inflammatory mediators. Apigenin was found to reduce phospho-IκB-α and suppress the activation and nuclear translocation of NF-κB, resulting in its protective effects in myocardial I/R injury.

In cardiomyocyte cell culture studies, cardiomyocytes exposed to OGD injury mimic the I/R process; however, pretreatment with apigenin (6 µM) has been shown to promote cell viability and inhibit apoptosis [46]. Apigenin was assessed by Li et al. for its cardioprotective effects using an in vitro model: rat H9c2 cardiomyocytes cell line subjected to ischemia/hypoxia (I/H)-induced injury [47]. The administration of apigenin (1, 6, and 25 μM) suppressed pyroptosis and apoptosis stimulated by I/H injury in H9c2 cells in a dose-dependent manner. The release of proinflammatory cytokines was significantly diminished following treatment with apigenin, signifying its cardioprotective effects.

Additional studies have indicated other prosurvival signaling pathways, such as PI3K/Akt and AMPK signaling pathways, to be associated with the cardioprotective actions of apigenin [48,49]. Protein kinase C ε (PKCε), belonging to the family of novel PKCs, has been demonstrated to provide a significant protective effect during ischemia reperfusion injury [50]. The outcomes of the study conducted by Zhu et al. revealed that pretreatment with apigenin increases SOD activity, decreases MDA levels, alleviates oxidative stress, and reduces cell apoptosis by PKCε activation [50]. Apigenin treatment increased Nrf2 nuclear translocation and HO-1 expression. The cardioprotective role of apigenin mediated by the upregulation of JAK2-STAT3 pathway was confirmed by Wang and colleagues. Administration of apigenin downregulates miR-15b expression and upregulates JAK2, leading to the activation of JAK2-STAT3 pathway, thereby attenuating I/R injury and myocardial apoptosis [51]. In addition, Huang et al. demonstrated that pretreatment with apigenin had a protective effect on the myocardium with improved cardiac function in rats that underwent I/R injury through the mitochondrial pathway mediated by Notch1/Hes1 signaling [52]. Apigenin also protects against myocardial infarction-induced injury by regulating parkin-mediated mitochondrial autophagy [53].

### 4.4. Cardiovascular Complications of Diabetes

Diabetes mellitus considerably increases the risk for the development of cardiovascular disease. Diabetic cardiomyopathy is a major complication of diabetes mellitus and contributes to an increased disease mortality. Diabetic cardiomyopathy is characterized by ventricular hypertrophy, myocardial fibrosis, steatosis, and apoptosis in the myocardium [54].

Apigenin administration (100 mg/kg) in a mouse model of diabetic cardiomyopathy has been shown to suppress streptozotocin (STZ)-induced hyperglycemia, alleviate cardiac hypertrophy and improve cardiac function [55]. Treatment with apigenin enhanced the activity of antioxidative enzymes in cardiac tissue. Apigenin also inhibited the nuclear translocation of NF-κB, resulting in the decreased production of IL-1β, IL-6, and TNF-α. Similar results were also obtained with in vitro studies performed on cardiac tissue and H9c2 cardiomyocytes.

Apigenin was further evaluated by Mahajan et al. for the treatment of myocardial injury in diabetic rats [56]. The administration of isoproterenol (ISO) in STZ-induced diabetic rats was used to mimic diabetes-associated acute MI. Apigenin was found to prevent hemodynamic changes and restore left ventricular function. Treatment with apigenin maintained endogenous antioxidants, restored a balanced redox status, and reduced apoptosis as well as lipid peroxidation in the myocardium. Apigenin also increased PPAR-γ expression. All these synergistically contribute to cardio protection against isoproterenol-induced myocardial injury. Apigenin mediates its protective effects through PPAR-γ pathway, and its effects were completely abolished in the presence of PPAR-γ antagonist.

Qin et al. further demonstrated the protective actions of apigenin against high glucose-induced endothelial dysfunction through the inhibition of protein kinase C βII (PKCβII) phosphorylation, reducing ROS production and apoptosis. Increase in eNOS activity also restored NO production [57]. Endothelial dysfunction is a chronic inflammatory process and plays a key role in the development of vascular complications of diabetes. Studies in HUVECs and HAECs exhibited improved cellular viability with apigenin treatment. Treatment with apigenin enhances the activation of the PI3K/Akt signaling pathway, which promotes endothelial cell survival and NO production through the activation of eNOS. Apigenin further suppressed the activation of NF-κB in HUVECs exposed to high glucose, leading to the inhibition of inflammation, adhesion, and chemotaxis.

### 4.5. Hypertension

Hypertension increases the morbidity and mortality of cardiovascular and kidney diseases [58]. The sympathetic nervous system plays a central role in the regulation of blood pressure and hypertension-induced cardiac hypertrophy. Inflammation and oxidative stress play an important role in the progression of hypertension [59].

Gao et al. showed improvement in hypertension and cardiac hypertrophy by the bilateral hypothalamic paraventricular nucleus (PVN) infusion of apigenin. Spontaneously hypertensive rats were treated with apigenin (20 μg/h) for 28 days via osmotic minipumps [60]. Apigenin infusion reduced the mean arterial pressure (MAP) and heart rate and attenuated cardiac hypertrophy and fibrosis in SHR rats. PVN infusion with apigenin decreased oxidative stress and improved antioxidant activity through the regulation of ROS generation and inflammation. Apigenin treatment attenuated the levels of proinflammatory cytokines, whereas PVN levels of interleukin 10 (IL-10) and SOD were increased. The outcomes of the study showed that apigenin plays a beneficial role in hypertension and related cardiac hypertrophy.

Apigenin has shown promising results in pulmonary hypertension, and it inhibits pulmonary artery smooth muscle cell proliferation (PASMCs) [61]. Exposure to chronic hypoxia was used to establish a rodent model of pulmonary hypertension. Apigenin (50 and 100 mg/kg, intragastric) prevented the progress of pulmonary hypertension, right ventricular hypertrophy, and pulmonary arterial remodeling. Treatment with apigenin induces apoptosis in hypoxic PASMCs via a mitochondria-dependent pathway. K+ channel impairment (KV1.5 and KV2.1) is observed in experimental pulmonary hypertension, resulting in an augmented influx of Ca^2+^, leading to the constriction and proliferation of PASMCs. Apigenin induces KV1.5 and modulates apoptosis in PASMCs. Furthermore, it was found that apigenin mitigates pulmonary hypertension via inhibiting the hypoxia-inducible factor, the HIF-1α-KV1.5 channel pathway in PASMC.

Apigenin also exerted antihypertensive action in a rat model of arterial hypertension induced by chronic administration (6 weeks) of L-NAME, a nitric oxide synthesis inhibitor [62]. The antihypertensive effect was complemented by a decrease in sodium retention. The increased production of NO enhanced the vasodilator ability. Apigenin treatment also produced favorable changes in the histopathological parameters in the heart and kidney. In another study, apigenin was found to improve aortic vascular relaxation in SHR rats [63].

Apigenin exerted significant improvement in endothelium-dependent relaxation in DOCA-salt hypertensive rats. In addition to improvement in kidney function, a reduction in elevated blood pressure was observed. This was supplemented by decreased expression of the transforming growth factor-β1 (TGF-β1)/Smad2/3 signaling pathway. Apigenin exerts a protective role in hypertension-induced renal fibrosis through the transient receptor potential vanilloid 4, TRPV4-mediated activation of the AMPK/SIRT1 pathway, which results in the inhibition of the TGF-β1/Smad2/3 signaling pathway [64].

### 4.6. Drug-Induced Cardiotoxicity and Cardiomyopathy

Doxorubicin, or Adriamycin, is an anthracycline antibiotic widely used as a chemotherapeutic agent for breast cancer, acute leukemia, and Hodgkin’s lymphomas [65]. However, cardiotoxicity limits its clinical usage. Apigenin administration has been shown to reduce cardiac injury and attenuate events associated with doxorubicin-induced cardiotoxicity. In doxorubicin-treated rats, the levels of AST, LDH, and CK were elevated, but these effects were ameliorated with apigenin (100 mg/kg, p.o.) treatment [66]. Doxorubicin induced oxidative stress in myocardial cells through excessive ROS production and NADPH oxidase activation. Redox defense systems and the Nrf2/HO-1 signaling pathway were weakened with doxorubicin treatment. Doxorubicin activated MAP kinases and NF-κB while the PI3K/Akt/mTOR signaling pathway was impaired. The administration of apigenin (20 μM) significantly diminished doxorubicin-induced oxidative stress and apoptosis in myocardial cells. Apigenin upregulated the PI3K/Akt/mTOR pathway, promoting cell survival while reciprocating p38/JNK/p53 MAP kinase and NF-κB signaling, leading to the attenuation of the cardiotoxic effects of doxorubicin. Apigenin further enhanced Nrf2 signaling, thereby improving the cellular redox defense system.

Zare et al. further investigated apigenin for potential cardio protective effects. The intragastric administration of apigenin (25 mg/kg) attenuated myocardial damage. Significant improvements were also observed in cardiac functional parameters [67]. Serum levels of cardiac and liver injury biomarkers as well as apoptosis were decreased with apigenin administration. These results signify the cardioprotective action of apigenin against cardiotoxicity induced by doxorubicin, a chemotherapeutic agent widely used in numerous therapeutic regimens for the treatment of carcinoma, sarcoma, lymphoma, and leukemia.

Different from other chemotherapeutic agents, isoproterenol is a non-selective β agonist. It causes DNA damage in cardiomyoblasts and induces apoptosis and mitochondrial dysfunction in cardiac cells, leading to severe adverse effects on the heart. Simultaneous administration of apigenin was, however, found to inhibit apoptosis in cardiomyoblasts [68]. Apigenin (10 µM) inhibited the expression of proinflammatory markers in isoproterenol-treated H9C2 cells. The inhibition of DNA damage and apoptosis were also observed. Apigenin treatment prevented isoproterenol-induced lipid peroxidation and improved the activities of SOD and GPx, further protecting against isoproterenol-induced oxidative injury.

### 4.7. Aortic Aneurysm

Abdominal aortic aneurysm is characterized by irreversible dilation of the abdominal aorta. Chronic vascular inflammation and loss of arterial wall integrity are distinctive features in abdominal aortic aneurysms. Apigenin prevented the formation of abdominal aortic aneurysms through the inhibition of NF-κB [69]. Apigenin administration (40 and 80 mg/kg, p.o.) in CaCl_2_-induced abdominal aortic aneurysm in rats reduced aneurysm formation and decreased pathological dilation of the aorta. Furthermore, treatment with apigenin suppressed the degradation of elastin in the aortic tissue, conserving the arterial structure as well as reducing the expression of MMP-2 and MMP-9, thereby attenuating inflammation in the aorta. Apigenin inhibits the phosphorylation of IκB, preventing the dissociation and nuclear translocation of NF-κB, attenuating its inflammatory actions and cascade.

### 4.8. Cardiac Hypertrophy

Cardiac hypertrophy is an adaptive mechanism of the heart due to constant pressure overload characterized by increased size of cardio myocytes. However, continuous hypertrophy may lead to heart failure. Apigenin inhibits the hypertrophy of cardiomyocytes through decreased expression of hypoxia-inducible factor-1 alpha (HIF-1α) with an increase in miR-122-5p. The activation of Smad signaling molecules by TGF-β1 results in the development of cardiac fibrosis. Apigenin suppressed the differentiation and synthesis of cardiac fibroblasts stimulated by TGF-β1. Administration of apigenin upregulated the expression of miR-122-5p and Smad7 while downregulating HIF-1α, Smad2/3, and p-Smad2/3 [70]. Smad signaling molecules are also negatively modulated by a few endogenous regulators. c-Ski (cellular Sloan–Kettering Institute) inhibits Smad2/3 and p-Smad2/3 and prevents the production of collagen as well as organ fibrosis. Apigenin inhibits the differentiation in cardiac fibroblasts induced by TGF-β1 due to a decrease in miR-155-5p expression and the consequent upregulation of c-Ski expression, resulting in the inhibition of Smad2/3 and p-Smad2/3 [71].

## 5. Pharmacokinetics and Toxicity of Apigenin

The absorption, distribution, metabolism, and excretion of apigenin has not yet been well-characterized in humans. Available studies from animal models bring forward the concerns with oral absorption and poor bioavailability of apigenin. Numerous efforts have been taken in the past few years to improve the pharmacokinetics characteristics and formulation strategy using novel carriers of drug delivery and development for enhancing its bioavailability. The pharmacokinetics and drug–drug interactions was recently reviewed by Tang et al. [72]

As it belongs to the BCS II class, it exhibits poor systemic bioavailability, due to its low lipid and water solubility; ingested apigenin is either excreted unabsorbed or rapidly metabolized after absorption. It is subject to sulfation and glucuronidation in vivo and is present in blood and tissues in the form of glucuronide or sulfate conjugates, which are excreted via the urine and feces. Apigenin has been reported to inhibit P-gp and CYP3A4 and thus may be involved in drug–drug interactions [72,73]. However, further studies are warranted to establish drug–drug or drug–food interactions.

Regarding toxicity, apigenin has been found to be a safer molecule in the doses studied for therapeutic and preventive efficacy in preclinical models. Reports regarding the adverse metabolic reactions of apigenin are very few; consequently, its dietary consumption is recommended [5]. Considering the negligible dietary safety and time-tested usage in humans, the molecule could be considered safe for the doses studied in preclinical models and humans. However, extensive regulatory toxicology studies are yet needed to establish its safety in human studies. Apigenin has been reported to induce hepatocyte degeneration and toxicity, mild sedation, and muscle relaxation in murine models at very high doses (100 and 200 mg/kg) [6]. However, chamomile, a rich source of apigenin on widespread use, has not been linked to clinically apparent liver injury.

## 6. Conclusions and Future Perspectives

Apigenin is a multimodal nutraceutical with therapeutic applications in several chronic diseases, such as cancer, Alzheimer’s disease, depression, diabetes, and cardiovascular disorders. Apigenin as a naturally derived bioactive molecule is greatly supported by preclinical studies to provide a powerful cardioprotective effect, supporting its development as a drug product for clinical translation. Its availability as a nutraceutical and utilization as a non-prescription dietary supplement also supports its safety profile in humans. However, extensive clinical trials are required before making recommendations for human usage. Studies standardizing these therapeutically beneficial effects, especially with respect to establishing a reproducible dose–response and dose–safety profile, are currently lacking and require further investigations.

Furthermore, apigenin has been shown to be unstable and poorly absorbed in the GI tract on oral administration. Thus, formulating an oral dosage form enhancing its stability and bioavailability, and supporting its therapeutic advantages in vivo are also necessary before being evaluated in clinical studies and for its translation into a clinically acceptable option for therapy.

## Figures and Tables

**Table 1:** Dietary sources of apigenin *.

Source (s)	Glycoside	Quantity (mg/100 g or mg/100 mL)
Olive oil, extra virgin	Apigenin aglycone	1.17
Common sage, fresh	Apigenin aglycone	2.40
Italian oregano, fresh	Apigenin aglycone	3.50
Rosemary, fresh	Apigenin aglycone	0.55
Buckwheat, whole grain flour	Apigenin 6-C-glucoside (Isovitexin)	0.90
Common wheat, whole grain flour	Apigenin 6,8-C-arabinoside-C-glucoside	30.47
Lentils, whole, raw	Apigenin 7-O-glucoside	0.18
Globe artichoke, heads, raw	Apigenin 7-O-glucuronide	7.40
Orange, pure juice	Apigenin 6,8-di-C-glucoside (Vicenin 2)	5.53
Celery (Seeds)	Apigenin 7-O-apiosyl-glucoside (Apiin)	111.00
Celery (Leaves)	Apigenin 7-O-apiosyl-glucoside (Apiin)	8.37
Parsley	Apigenin 7-O-apiosyl-glucoside	4503.50 (dried)
		215.46 (fresh)
Kumquats	Apigenin 7-O-neohesperidoside	21.87

* These data were compiled from http://www.phenol-explorer.eu [9] (accessed on 5 March 2022) and USDA Database for the flavonoid content of selected foods, Release 3.3 (March 2018) (https://www.ars.usda.gov) [10] (accessed on 5 March 2022).

## Data Availability

This manuscript is a review and most of the studies referred to herein this article have been appropriately cited in the manuscript.

## Abbreviations

ABCA1	ATP-binding cassette A1
BCS	Biopharmaceutics classification system
GSK-3β	Glycogen synthase kinase-3β
HBMVEC	Human brain microvascular endothelial cells
HAEC	Human aortic endothelial cells
HUVEC	Human umbilical vein endothelial cells
HO-1	Heme oxygenase-1
HMGCR	3-hydroxy-3-methylglutaryl coenzyme A reductase
ICAM-1	Intercellular adhesion molecule-1
JAK2-STAT3	Janus kinase 2/signal transducer and activator of transcription
LDH	Lactate dehydrogenase
MCP-1	Monocyte chemoattractant protein-1
MAPK	Mitogen-activated protein kinase
NF-κB	Nuclear factor-kappa B
Nrf2	Nuclear factor-erythroid factor 2-related factor 2
Npc1l1	Niemann-Pick C1-like 1
NQO1	NAD(P)H quinone dehydrogenase 1
OGD-R	Oxygen and glucose deprivation and re-oxygenation
PAI-2	Plasminogen activator inhibitor-2
SREBP-2	Sterol regulatory element binding protein-2
TNFα	Tumor necrosis factor-α
TLR	Toll like receptor
VEGF	Vascular endothelial growth factor
VCAM-1	Vascular cell adhesion molecule-1
